# One Health security lessons from a year-long webinar series on international COVID-19 response

**DOI:** 10.1186/s42522-022-00071-0

**Published:** 2022-10-08

**Authors:** Caroline R. M. Kennedy, Yuri Bruinen de Bruin, Anne-Sophie Lequarré, Rebecca T. Ackerman, Jill Luster, Tiffany M. Tsang, Kari D. McInturff, Cassandra P. Carter, Richard Pilch

**Affiliations:** 1 The MITRE Corporation, McLean, Virginia USA; 2 Joint Research Centre, Directorate for Space, European Commission, Security, and Migration, Geel, Belgium and Ispra Italy; 3 Federal Bureau of Investigation, Washington DC, USA; 4 Middlebury Institute of International Studies, James Martin Centre for Nonproliferation Studies, Monterey, California USA

**Keywords:** COVID-19, One health security, Pandemic preparedness, Response, Information sharing

## Abstract

Following the principles outlined by the Global Outbreak Alert and Response Network, the Federal Bureau of Investigation’s International Biosecurity and Prevention Forum, the European Commission’s Joint Research Centre, and the Middlebury Institute of International Studies’ James Martin Center for Nonproliferation Studies cohosted a webinar series from April 2020 to January 2021 on COVID-19 management across Africa, Europe, and North America. We provide here an overview of the webinar series and discuss how lessons learned during the COVID-19 pandemic and debated during the webinars can be used to bridge One Health with biological threat-driven health security. This report can be used to inform recommendations for future One Health security approaches to strengthen global capacity and multidisciplinary cooperation.

**Supplementary Information:**

The online version contains supplementary material available at 10.1186/s42522-022-00071-0.

## Background

The COVID-19 pandemic has provided hard-earned yet invaluable lessons for global One Health security. One Health security is defined as the collaborative effort of public health, veterinary, environmental, and law enforcement disciplines working locally, nationally, and globally, to attain optimal health for people, animals, and the environment against natural, accidental, or nefarious biological threats. [1]. While One Health security strategies and capacities have been promoted by international organizations such as the World Health Organization (WHO), Food and Agriculture Organization (FAO), World Organization for Animal Health (WOAH), and the United Nations Environment Programme (UNEP) – known together as the Quadripartite [2] – implementation at the national level requires commitment and resources that are not consistent across member states [3].

In response to COVID-19, countries have applied geographically and culturally tailored hierarchical control strategies comprising measures aimed at reduction and control, if not elimination, of the pandemic [4]. Though most of these strategies share common goals, implementation has varied widely and led numerous countries to chaotic and disruptive situations and, in some cases, violence [5–7]. On a country-by-country basis, implementation has been further challenged by factors such as interconnected and highly mobile populations, socio-economic and socio-political drivers for enforcement, and misinformation and disinformation [8, 9]. Consequently, differing approaches to controlling the current pandemic and its socio-economic consequences have resulted in varying levels of success [10].

Relevant to this issue, the Global Outbreak Alert and Response Network (GOARN) identified strengthening national capacity and regional networks through electronic communication, regular meetings, and joint projects as key activities for bridging One Health and health security [11, 12]. Relatedly, convening multidisciplinary stakeholders from national and regional levels and incorporating corresponding lessons learned into international One Health security strategies and their national-level implementation is critical to preparing for future biological threats.

The webinar series aimed to enable just that. Through the series, we established connections between One Health and health security experts across three continents and leveraged those connections to share information and best practices with the goal of enabling global capacity building and multidisciplinary cooperation vis-a-vis COVID-19 preparedness and response. Participants discussed their local, regional, national, and international experiences and, in doing so, informed recommendations on prevention, detection, and response to One Health security challenges like COVID-19. This paper reviews lessons learned from the series of six One Health security webinars held during the first year of the COVID-19 pandemic (2020–2021) and with the goal of informing said One Health security recommendations.

### Webinar series overview

**Fig. 1:**
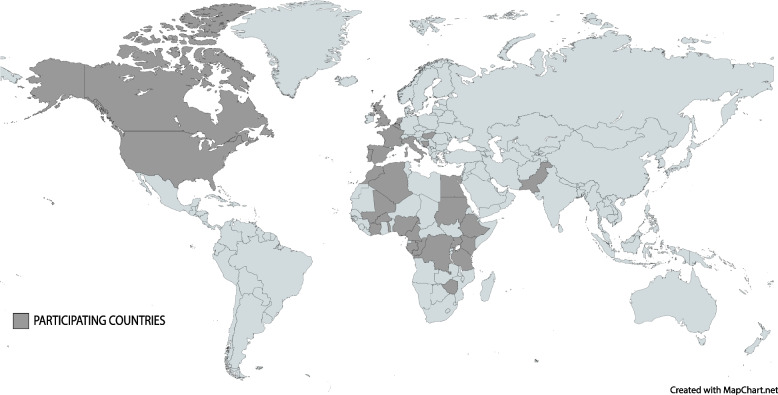
Map of countries participating in the webinar series

For much of 2019, the Federal Bureau of Investigation’s (FBI) International Biosecurity and Prevention Forum (IBPF); the European Commission’s Joint Research Centre (JRC), Directorate of Space, Security and Migration; and the Middlebury Institute of International Studies’ James Martin Center for Nonproliferation Studies (CNS) had been planning a March 2020 One Health Security Symposium for African Countries in Northern Italy. When the Milan area suffered the first major COVID-19 outbreak outside of China just days before the symposium, those plans changed. Like the rest of the world, we had a responsibility to limit the spread of the COVID-19 and that meant staying home. But unlike the rest of the world, we had built a network of over 100 global health and health security professionals across Africa, Europe, and the Americas (Fig. 1). That meant we had another responsibility—building a global platform facilitating the sharing of information, best practices, and lessons learned across the network to better manage the pandemic. The result was a year-long webinar series during which top experts discussed key aspects of the international COVID-19 response and engaged in open dialogue with their peers. We held a total of six webinars (Table 1) that retrospectively tell the story of the global community’s evolving understanding and management of COVID-19.

**Table 1:** Listing of webinars

Date	Title	Speakers
07 April 2020	The COVID-19 Outbreak in Italy: Crisis Management and Medical Experiences	Dr. Leonardo Radicci, Logistic Coordinator for COVID-19 Outbreak Response, EMERGENCY NGO Dr. Stefan Mandić-Rajčević, Postdoc Fellow, University of Milano Dr. Federica Masci, Postdoc Fellow, University of Milano
17 June 2020	The COVID-19 Response in Africa and Risk Mitigation Measures implemented in Europe	Mr. Donewell Bangure, Epidemiologist, Africa Centres for Disease Control and Prevention Dr. Chanceline Bilounga Ndongo, Senior Officer, Cameroon Ministry of Public Health Dr. Yuri Bruinen de Bruin, Science and Policy Officer, European Commission, Joint Research Centre Dr. Anne-Sophie Lequarré, Scientific Project Officer, European Commission, Joint Research Centre
22 July 2020	Global COVID-19 Modeling	Dr. Christopher Murray, Institute Director of the Institute for Health Metrics and Evaluation, University of Washington
26 August 2020	COVID-19 Epidemiology and Evolution	Dr. Satish Pillai, Deputy Director of the Division of Preparedness and Emerging Infections, US Centers for Disease Control and Prevention Dr. Paul Jackson, Affiliate, Stanford University, Center for International Security and Cooperation, Adjunct Professor Middlebury Institute of International Studies Rebecca Ackerman, Management and Program Analyst, Federal Bureau of Investigation
23 September 2020	COVID-19 Environmental Surveillance and Risk Communication	Dr. Bernd Gawlik, Scientific Project Manager, European Commission, Joint Research Centre Dr. Suvajee Good, Regional Advisor for Health Promotion and Social Determinants of Health, World Health Organization Ms. Liliane Luwaga, Risk Communication Consultant, World Health Organization
27 January 2021	What Comes Next? Security Implications for a Post-COVID World	Dr. Rob de Wijk, Founder, The Hague Centre for Strategic Studies Dr. Nonye Welle, Medical Director, Police Hospital Garki-Abuja, Nigeria Dr. Gary Ackerman, Associate Professor, University at Albany, College of Emergency Preparedness, Homeland Security, and Cybersecurity

## Recommendations for enhancing prevention, detection, and response as part of a One Health security approach

The webinar series provided participants with an opportunity to communicate and share information on challenges, approaches, and outcomes on how their communities have been responding to the COVID-19 pandemic. In our review of concepts discussed during the webinars (Supplemental File 1), we identified common themes (e.g., contact tracing, public health messaging), as well as some gaps (e.g., disease transmission questions to which there were not yet answers). Informed by these themes and gaps, we developed high-level recommendations for enhancing prevention, detection, and response in anticipation of the next One Health security challenge.

### Prevention

#### Minimize risks at the animal-human interface

Animal reservoirs harbor an unknown number of viruses that may cause disease in naïve human populations if exposed. The animal-human interface where such exposure occurs is part of the “epidemiological triangle”—the balance between a disease agent, a human host, and the environment. The disease agent and human host come into contact when the human populations extend into animal reservoirs, or disease agents extend into human populations; environmental factors play a role in both. Human populations may extend into areas where the disease agent resides in animal reservoirs due to wildlife trade, deforestation, industrial farming, and other ecologically disruptive activities. Disease agents may extend into human populations due to animal reservoir overgrowth, vector population overgrowth (e.g., ticks, fleas) that may follow a loss in biodiversity, interspecies spillover, and other mechanisms. Short-term meteorological shifts and longer-term climate shifts poses additional human-caused environmental risks.

While One Health security experts have long-recognized the need to minimize risks at the animal-human interface, reactionary measures such as temporary closure of wet markets have proven insufficient to prevent subsequent outbreaks. A global shift in behavioral norms that provide sustainable alternatives to both the supply and demand of higher-risk activities, including affordable and culturally accepted consumer alternatives, is required. Additional measures to reduce risk at the animal-human interface include active surveillance at high-risk areas for zoonotic disease transmission, such as routine environmental sampling in wet markets or in dead wild animals; improved sanitary practices in these locations and populations; consumer education on safe handling and consumption of animal products; and increased efforts to combat the illegal wildlife trade. The last measure may be more palatable to governments if the nexus between illegal wildlife trade and transnational crime organizations and terrorist groups is emphasized. [13].

#### Minimize laboratory science, safety, and security risks

An increasing number of laboratories around the world are performing research involving highly dangerous pathogens that may lead to accidental or deliberate biological events on the scale of COVID-19. There is no single international laboratory registration and accreditation system, incident reporting system, or formal safety auditing program in accordance with the International Organization for Standardization’s biological risk management standard 35001:2019 [14] or the WHO’s Laboratory Biosafety Manual (4th edition, 2020), [15] nor are there corresponding internationally recognized laboratory security guidelines governing personnel reliability, insider threat detection, and material accountability [16]. Accompanying this trend is an insufficient number of laboratory staff who have current and comprehensive biosafety and biosecurity training commensurate with the type of laboratory research they are conducting [17]. Additional risks include the lack of international peer review, approval, and oversight of certain types of high-risk research that pose unique risks to global communities.

While it may not be feasible or necessarily desirable to develop a single international laboratory science, safety, and security assurance system, it may be advantageous to provide countries with a single resource for laboratory science, safety, and security best practices. This could be in the form of a multilateral “responsible laboratory operations” regime operating under the auspices of the Quadripartite that centralizes, updates, and regionally contextualizes laboratory science, safety, and security information and resources for member states. Such a mechanism could be created under future International Health Regulations revisions or the WHO’s proposed pandemic treaty [18, 19] currently in development and potentially be granted coordination and information sharing authorities during public health emergencies of international concern. A stronger adherence to the Biological and Toxin Weapons Convention could also boost the endorsement of biosafety and biosecurity standards and help building a robust international health security architecture.

#### Continue to foster intersectoral and international relationships

Continued efforts to de-silo One Health security sectors are imperative to improving health security, preparedness, and prevention. For example, the Joint Criminal-Epidemiologic Investigations Program, [20] a training partnership between the FBI and Centers for Disease Control and Prevention (CDC), brings together public health and law enforcement personnel from the same region to learn how to investigate, in partnership, unusual or unexpected outbreaks. Because anomalous outbreaks could result from natural, accidental, or nefarious causes, parallel and intersecting investigations contribute to appropriate response activities and attribution determination. Applying this same format, the FBI collaborates with the United States Department of Agriculture (USDA) Animal Plant Health Inspection Service (APHIS) for the FBI/USDA-APHIS’s Animal-Plant Health Joint Criminal-Epidemiological Investigations course, in recognition that anomalous outbreaks are not limited to humans [21]. The FBI has further established a Weapons of Mass Destruction Coordinator (WMDC) role at each of the 56 FBI field offices to cover chemical, biological, radiological, nuclear, and explosives investigations at the regional level alongside public health and local law enforcement and to help identify overt and covert biological incidents [22]. In the European Union (EU), Mediterranean and Black Sea Field Epidemiology Training Programme Network (MediPIET) [23] and Europol, in partnership with the European Centre for Disease Prevention & Control, conduct joint trainings with law enforcement, public health, and civil protection authorities [24]. By fostering intersectoral relationships before an outbreak or biological incident, response officials reduce the chances of insufficient communication resulting in missed warning signs.

Equally important to effective prevention measures are international cooperation and collaboration. Programs that foster international relationships are useful in building the connections that can mitigate the effects of a One Health security event. For example, the FBI’s International Biosecurity and Prevention Forum is a unique tool that brings together leading experts from the health and security communities to share experiences, expertise, and best practices on key biosecurity and bioterrorism prevention issues. For years preceding COVID-19 and throughout the pandemic, the International Biosecurity and Prevention Forum had maintained an international member-driven website and resources promoting international collaboration leading to symposium, trainings, and exercises aimed at preventing and responding to One Health security incidents. Activities such as the ones conducted by INTERPOL [25] or through a recent partnership between WOAH, FAO, and INTERPOL [26] build global resilience against animal health emergencies following malevolent acts. The EU Centres of Excellence on Chemical, Biological, Radiological, and Nuclear Risk Mitigation [27] are also stimulating similar intersectoral cooperative relationships across its transnational network.

### Detection

#### Increase capability and capacity for genomic sequencing and information-sharing

Researchers’ early reporting of the SARS-CoV-2 genome [28–30] accelerated development of targeted diagnostics, vaccines, and treatments. As the pandemic progresses, international collaboration on the rapid sharing of genomic information via global on-line platforms [31] remains particularly helpful for genomic surveillance and tracking transmission patterns and emerging variants. Along with the accumulation of increasing genomic sequencing data, artificial intelligence/machine learning approaches can be applied to predict the emergence of variants that might become a concern. However, there are limitations to more widespread genomic sequencing and information sharing, including insufficient access to high throughput sequencing technologies, particularly in low resource settings, [32] and concerns surrounding properly crediting researchers for their sequenced genomes [33]. Efforts should be made to explore alternative high throughput sequencing methodologies that require less operator training and fewer resources, establish centralized locations to conduct all the high throughput sequencing for any given low resource region, [34] and develop institutional policies that balance open access to raw genomic data with benefit-sharing mechanisms [35].

#### Increase testing and contact tracing capacity and innovation

COVID-19 exposed diagnostic testing and contact tracing shortfalls that limited the ability of some nations to rapidly identify and isolate cases and to trace and quarantine close contacts in order to disrupt the chain of transmission. Innovative measures to expand testing and contact tracing capacities include rapid antigen detection tests in occupational settings [36] or settings with high COVID-19 prevalence, subpopulation testing such as city- or institute-level sewage surveillance [37] and secure contact tracing applications (e.g., mobile device applications) [38]. To maximize testing and training capacities, regions should select methods (e.g., rapid antigen vs. reverse transcription polymerase chain reaction, pooled testing vs. individual testing) based on current prevalence rates, desired outcomes, and available resources. To this purpose, there may be utility in development of a decision matrix to guide regions in designing and deploying a surveillance strategy that fits their needs.

#### Take a geospatial technology-driven approach to early warning systems

Geospatially driven approaches may be used to build early warning systems that detect potential for outbreaks or the very early stages of an outbreak. Currently, early stages of outbreaks are typically detected through event-based surveillance systems like the Program for Monitoring Emerging Diseases (ProMED), a service in which health experts around the world send in reports of plant, animal, and human diseases of either known or unknown etiology. ProMED reported a set of symptoms consistent with those now known to be caused by SARS-CoV-2 on 30 December 2019, one day before official reports were released [39]. To supplement event-based surveillance systems, organizations have experimented with automated detection of disease occurrence and pathogen emergence but are limited in part by poor data quality, interoperable data, or incomplete/nonexistent data. Though there are limitations to automated disease and pathogen detection, continued efforts are needed. An automated surveillance system would provide an invaluable service and give communities additional time to prepare for or quell an impending outbreak. If built, an ideal system would be global in nature and ingest various geospatial and non-geospatial data sources, to include physiological and symptomatic data from human, animal, and agricultural health systems; meteorological and climate data; deforestation patterns; pathogen surveillance data from environmental biosensors; and data on population mobility (e.g., air and ground transportation patterns), population density and demography reports, economic indicators like Gross Domestic Product, and social media. Such a system could be used in parallel with more focused Geographic Information Systems to support public health planning and decision-making [40].

### Response

#### Add to body of research about the role misinformation and disinformation have in public health response efforts and how to counteract them

COVID-19 is not only the first pandemic most of us have experienced, but also the first one experienced in the digital age. This has enabled global citizens more rapid access to situational updates from trusted medical advisors, but it has also enabled rampant proliferation of misinformation and disinformation. Misinformation and disinformation are similar but have an important difference, intent. With misinformation, there is no intent to spread incorrect information; with disinformation, there is [41]. Dissemination of misinformation and disinformation was widespread during the COVID-19 pandemic. For example, one article estimated that 46% of the population in Britain were exposed to fake news about the coronavirus and that 25% of the top watched coronavirus videos on YouTube contained misleading information of some kind [42]. The result is increased distrust in health care professionals [43] and potentially even democratic governance disruptions [44]. In One Health security response plans moving forward, officials must account for the effects these information types will have on community uptake of official messaging and develop plans to counteract the harm propagated by misinformation and disinformation in the future. More research into how communities consume health and security information in the digital age and research grounded in cognitive psychology, behavioral economics, and applied epistemology is needed to ensure mitigation strategies are effective.

#### Develop scalable solutions for healthcare supply chain vulnerabilities

In the global response to the COVID-19 pandemic, there were many instances of healthcare supply chain shortages that affected and hindered response efforts. At the start of the pandemic, the nasopharyngeal swabs needed to conduct COVID-19 tests were only produced by two manufacturers worldwide, [45] and shortages of ventilators, respirators, surgical masks, and various other types of personal protective equipment threatened to derail response efforts while cases were on the rise [46]. Supply chain vulnerabilities can arise for many different reasons, but during this pandemic, we have seen shortages stemming from overreliance on one or two companies to produce an item and production that is overly concentrated in one geographical region with no means for transporting products across borders during lockdowns.

Though we do not know how the next pandemic will spread, we have a sense of what types of pathogens have pandemic potential, as well as their transmission routes. Based on that information, governments could develop strategic autonomy plans at national or regional levels and assess how they might redirect various industries (e.g., car manufacturing, distilleries) to meet urgent supply chain needs.

#### Provide front line law enforcement officers with training on safe response during public health emergencies

Law enforcement officers fulfill many roles in a community, and these roles multiply during a public health emergency. For example, during a public health lockdown, jurisdictions may call on their law enforcement officers to assist with overseeing movement of people or goods across borders. Law enforcement officers may have to enforce face mask mandates or gathering restrictions, or be asked to accompany shipments of therapeutics, medical supplies, or biological specimens [47]. They may also be needed to intervene when healthcare workers or facilities receive threats. Regardless of their precise roles during a public health emergency, law enforcement officers must be given the training and resources needed to maintain their safety and that of the public. This training should include information about the pathogen and its associated disease (e.g., symptoms, transmission routes) if the public health emergency is infectious in nature [48]. Law enforcement officers should be informed about non-pharmaceutical interventions (e.g., mask wearing, social distancing) they can employ to reduce risk of infection and provided with personal protective equipment commensurate to the public health emergency. Lastly, law enforcement officers should be kept apprised of any crime trends stemming from the public health emergency (e.g., increase in domestic violence, threats to healthcare systems/facilities); changing crime trends may result in increases of specific types of calls, and officers should be prepared for their duties to shift accordingly. This is a best practice employed by the FBI through their development of Public Safety Awareness Reports, which share information on crime trends and considerations for law enforcement while working during a public health emergency.

## Discussion

The webinar series focused on approaches to better understand and respond to COVID-19 to reduce its spread. At the heart of the dialogue was diversity of thought, experience, and expertise from around the world, as well as access to an open forum for information exchange. Through something as simple as a roughly bimonthly webinar, researchers, clinicians, law enforcement, and policy makers working to mitigate the effects of COVID-19 in their communities were able to connect with each other to bridge One Health and health security. In doing so, each participant’s invaluable perspectives informed One Health security activities of the group and created a platform for transnational situational awareness to identify and implement globally recognized and prioritized One Health security actions [3].

An important aspect to the transnational focus of both the canceled in-person event and executed webinar series is the flow of information we worked to foster between participants from high-income countries (HIC) and low- and middle-income countries (LMIC). The in-person event and webinar series were invite-only to ensure our numbers were small enough so as to encourage conversation but also to balance attendee country of work, sector (e.g., human, animal, and/or environmental health, law enforcement, military, policy, etc.), and gender—in short, to promote diversity and innovative thinking and problem solving [49]. Regarding our consideration of country of work, just as Global South-to-Global South health security mentorships have been shown to improve laboratory biosafety and biosecurity outcomes and policy development, [50]. we surmised that LMIC-to-LMIC One Heath security professional connections could improve implementation of solutions to One Health security challenges shared across collocated regions and nations. In addition to the information flow between LMIC participants, we also wanted to encourage a bidirectional exchange between HIC and LMIC participants. To this point, we identified webinar speakers from HIC and LMIC countries to present during shared sessions and used moderated discussions at the end of presentations to encourage information flow between presenters and participants from both HICs and LMICs.

### Limitations

Though the webinar series afforded participants the opportunity to share information on COVID-19 response in their regions, the originally planned event was meant to take place in person with an agenda that focused heavily on small group discussion and brainstorming activities to encourage active participation from all attending. Pivoting to a virtual event was necessary, but it limited the organic connections and conversations that can contribute to innovative thinking and problem solving. Our team worked to encourage this by promoting discussion in the webinars’ chats and post-webinar emails exchanges, as well as sending surveys to participants after the webinars to solicit feedback more directly.

**Fig. 2:**
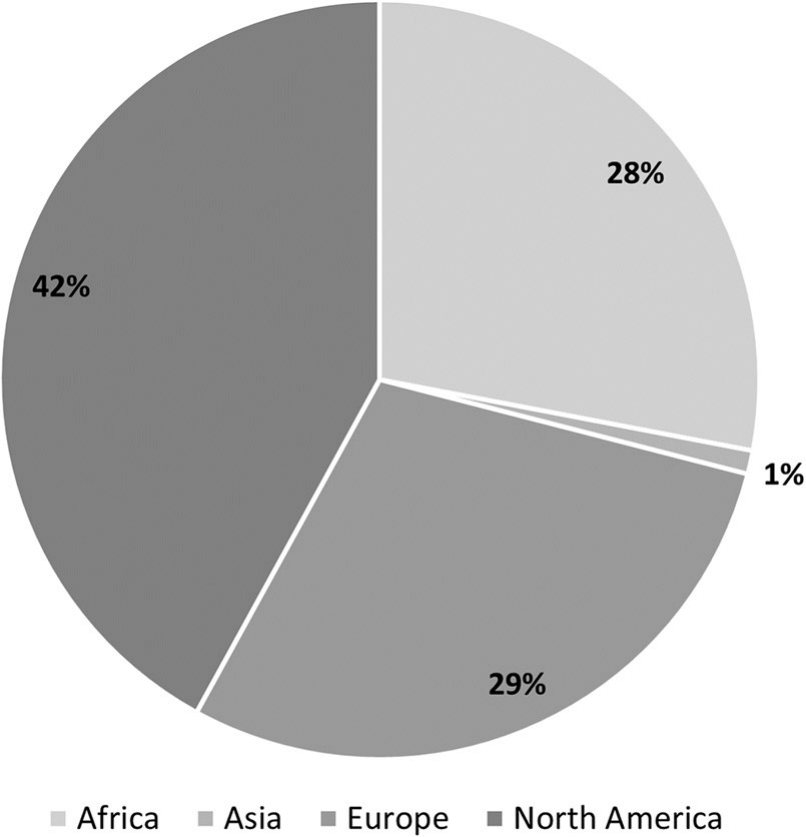
Breakdown of participants by continent in which they work

The webinar format resulted in other challenges that may have hindered communication and freer exchange of ideas due to technical complications. Internet connections were inconsistent at times, which occasionally caused choppy presentations and some missed content. However, with the speakers’ and participants’ permission, we recorded the videos and posted them on the IBPF website so they could be rewatched, and speakers made themselves available to participants afterwards for questions to fill in any gaps technical glitches may have caused. Additionally, on two occasions participants did not enter their information upon joining the webinar; as such, we did not know the countries in which they worked and were not able to add those countries to Figs. 1 and 2. As such, it is possible the figures are missing two additional countries.

## Conclusions

Overall, we expect the greatest return on investment in One Health security to come from preventing pandemics before they occur. Doing so will likely require enhancing global biological risk mitigation mechanisms by strengthening data standards, equipping national health systems with baseline data analysis capabilities, and creating sustainable and equitable data sharing agreements between nations. Equally important will be integrating economic considerations with One Health security goals so that if another pandemic were to occur, global supply chains – particularly those pertinent to healthcare – will be preserved. We recognize these are no easy tasks. Pandemics are global in nature and require coordinated global response to mitigate them, yet governance is largely national; as such, regional or national interests may not always align with international interests. Diplomacy, intergovernmental agreements like the proposed pandemic treaty, and global research collaborations can all be used as tools in the pandemic preparedness and response toolkit to encourage coalescence of One Health security action across governments worldwide.

## Supplementary Information

**Additional file 1.** 

## Data Availability

Not applicable.

## Abbreviations

WHO: World Health Organization
FAO: Food and Agriculture Organization
WOAH: World Organization for Animal Health
UNEP: United Nations Environment Programme
GOARN: Global Outbreak Alert and Response Network
FBI: Federal Bureau of Investigation
IBPF: International Biosecurity and Prevention Forum
JRC: Joint Research Centre
CNS: Center for Nonproliferation Studies
CDC: US Centers for Disease Control and Prevention
USDA: United States Department of Agriculture
APHIS: Animal lant Health Inspection Service
WMDC: Weapons of Mass Destruction Coordinator
EU: European Union
MediPIET: Mediterranean and Black Sea Field Epidemiology Training Programme Network
INTERPOL: International Criminal Police Organization
ProMed: Program for Monitoring Emerging Diseases
