# Supplementary material for: One Health security lessons from a year-long webinar series on international COVID-19 response
Source: One Health Outlook. 2022 Oct 8;4:15. doi: 10.1186/s42522-022-00071-0 (PMC9547628; doi:10.1186/s42522-022-00071-0)
Supplement: Supplementary file 1 — Additional file 1. [file 42522_2022_71_MOESM1_ESM.docx]

**Supplemental File 1**

**Webinar 1: The COVID-19 Outbreak in Italy: Crisis Management and Medical Experiences (7 April 2020)^[[1]](#endnote-1)^**

Outside of China, the Milan area of Northern Italy was one of the hardest hit regions in the earliest stages of the pandemic, such that first responders and hospitals were able to document and provide valuable lessons learned in the fight against COVID-19. At our first webinar, Dr. Leonardo Radicchi, a Logistics Coordinator for COVID-19 outbreak response, shared lessons from his experience during COVID-19 crisis management at Italy’s Bergamo Hospital at the University of Milan. Dr. Radicchi discussed infection control measures and gave an overview of how to compartmentalize people and equipment in healthcare facilities to reduce the spread of the virus. Dr. Stefan Mandić-Rajčević and Dr. Federica Masci then shared lessons from their medical experience during the Northern Italy surge, in particular lessons regarding best practices for improving occupational health and safety in hospitals. Additional topics of discussion included a universal COVID-19 case definition, reverse transcription polymerous chain reaction and false negatives, contact tracing, quarantining patients at home with family, personal protective equipment shortages among first responders/police and in Italian hospitals, patient triage, disposal of infectious waste, steps to take regarding deceased patients, protective immunity for recovered patients, and how COVID-19 impacts different demographics.

**Webinar 2: The COVID-19 Response in Africa (17 June 2020)^[[2]](#endnote-2)^**

# By 01 June 2020, the World Health Organization (WHO) COVID-19 dashboard had recorded 104,402 cumulative COVID-19 cases on the African continent, compared to 2.84 million in the Americas and 2.15 million in Europe.^[[3]](#endnote-3)^ The continent would experience a surge in confirmed cases to over one million by the end of August 2020, reflecting both increased incidence of disease and expanded testing capacity. At our second webinar, Mr. Donewell Bangure from the African Centres for Disease Control and Prevention discussed related laboratory support efforts of the African Centres for Disease Control and Prevention, including its Africa-wide "Partnership to Accelerate COVID-19 Testing" strategy. Dr. Chanceline Bilounga from Cameroon’s Ministry of Health then discussed Cameroon’s COVID-19 experience as a case study for national level prevention, detection, and response on the continent. Additionally, as a follow up to Webinar 1, the Joint Research Centre’s Dr. Yuri Bruinen de Bruin and Dr. Anne-Sophie Lequarré presented a COVID-19 Risk Mitigation Model applying risk management lessons from the European Union experience.^[[4]](#endnote-4)^ Additional topics of discussion included accuracy of early disease transmission models, influence of demography, predicting future spikes in COVID-19 cases, cost-benefit analysis of risk mitigation measures, increase testing capacities and quality control of COVID-19 tests, infection control at international borders, and lessons learned from non-African countries that have advanced further along the epidemiological curve.

**Webinar 3: Global COVID-19 Modeling (22 July 2020)^[[5]](#endnote-5)^**

Six months into the pandemic, predictive modeling had become a mainstay of national and international briefings on the state of the pandemic. Albeit imperfect, such forecasts – of both disease incidence and deaths – provided a trackable metric against which the impact of various interventions could be measured. In the United States, the forecasts most commonly presented during White House press briefings were developed by the University of Washington’s Institute for Health Metrics and Evaluation, which had recently updated its pandemic models for the African continent. At our third webinar, the Institute for Health Metrics and Evaluation Director Dr. Christopher Murray provided an overview of disease forecasting principles, including model history and development; key drivers of cases and deaths based on mandates, mobility, masks, and seasonality; transition models and the tendency to overestimate; alternate prediction scenarios;^[[6]](#endnote-6)^ and recommendations for model development. He then provided a user-focused demonstration of the Institute for Health Metrics and Evaluation’s Africa models using cumulative death projections in Sub-Saharan Africa, Mozambique, and Cameroon as case studies. Additional topics of discussion included model differences and which independent model offers the most predictive/accurate information; modeling mask mandates, sustained immunity, herd immunity, and super spreaders; the impact vaccine availability and efficacy will have on model predictions; the ability of models to detect changes in viral behavior due to mutation; best practices for communicating model predictions to policymakers and the general public; the long-term, global outlook of COVID-19; and specific transmission data trends in Burundi and Tanzania, where no public health restrictions (e.g., masks, physical distancing, limiting indoor gatherings, travel) had been mandated.

**Webinar 4: COVID-19 Epidemiology and Evolution (26 August 2020)^[[7]](#endnote-7)^**

By the end of August, considerable attention was still being paid to mass testing and contact tracing strategies; in addition, genomic analysis was providing an increasingly clear picture of circulating COVID-19 variants and their potential for emergence. In the next two months, SARS-CoV-2 variants B.1.1.7 (“UK variant”)^[[8]](#endnote-8)^ and 501Y.V2 (“South Africa variant”) would take hold.^[[9]](#endnote-9),^^[[10]](#endnote-10)^ At our fourth webinar, Dr. Satish Pillai from the US Centers for Disease Control and Prevention (CDC) reviewed the epidemiology of COVID-19, including observed COVID-19 transmission dynamics in the United States, the epidemiological role of the US public health system, and CDC response efforts during the pandemic. Following Dr. Pillai’s review, Dr. Paul Jackson of Stanford University described the genomic evolution of the virus, including function of different viral genes, the role of the spike protein and what genetic mutations might affect it, frequently seen mutations in sequenced strains of SARS-CoV-2, and the most common circulating SARS-CoV-2 variants in different populations and geographic areas. Additional topics of discussion included public health messaging and outreach targeted at specific groups, for example teenagers and young adults contributing to viral transmission; blood type as a risk factor for disease severity; testing and contact tracing strategies and limitations; the impact of viral mutations on test accuracy; the potential for reinfection by SARS-CoV-2; case fatality rates among different demographics; and treatment options. Lastly, Rebecca Ackerman from the Federal Bureau of Investigation (FBI) provided an overview of the International Biosecurity and Prevention Forum (IBPF) collaboration website as a valuable tool for international and inter-sectoral communication and information sharing across the one health security community.

**Webinar 5: COVID-19 Environmental Surveillance and Risk Communication (23 September 2020)^[[11]](#endnote-11)^**

In anticipation of a fall/winter surge in COVID-19 cases, our fifth webinar focused on approaches to expand surveillance and improve risk awareness and communication among at-risk populations and the general public. Our first speaker was Dr. Bernd Gawlik, a Program Manager at the European Commission’s Joint Research Centre, who reviewed advantages and limitations of sewage surveillance in European countries, including predictive value, cost-benefit analysis, and environmental sample collection and testing strategies (e.g., frequency, mapping, cost estimation). Next, Dr. Suvajee Good, WHO Regional Advisor for Health Promotion and Social Determinants of Health, and Ms. Liliane Luwaga, WHO Risk Communication Consultant, described risk communication strategies, misinformation campaigns, rumor tracking tools, and community engagement guidance and experiences. Additional topics of discussion included vaccine communication; unique challenges to communicating risks of COVID-19 versus past outbreaks; the speed of international attention; and misinformation/disinformation management.

**Webinar 6: What Comes Next? Security Implications for a Post-COVID World (27 January 2021)^[[12]](#endnote-12)^**

# Over a year into the pandemic, accelerated vaccine development had enabled vaccination of frontline healthcare workers and high-risk populations in many countries around the world, while it remained unclear when other countries would receive their first doses. For our sixth and final webinar, we therefore looked to the future, to discuss such topics as global supply chain vulnerabilities, equitable vaccine distribution, COVID-19’s impact on the international security landscape and preventing and preparing for the next pandemic. First, Dr. Rob de Wijk, Founder and Director of the Hague Centre for Strategic Studies, focused his remarks on learning from past incidents; international and geopolitical consequences; nationalism and supply chains; and acceleration of global powershifts. Next, Dr. Nonye Welle of the Police Hospital in Garki-Abuja, Nigeria, described the pandemic’s negative effects on almost all sectors of human endeavor including health, agriculture, tourism, trade, transportation, economies, sports, and the significant threat to safety. Finally, Dr. Gary Ackerman of the College of Emergency Preparedness, Homeland Security, and Cybersecurity, University at Albany, described the effect of the pandemic on Violent Non-State Actors, including reduced operational efficacy due to susceptibility to the coronavirus; operational frictions due to such factors as funding constraints and border closings; and the potential for expanded attacks in some parts of the world, as seen for example with Boko Haram and Ansar al-Sunna.

Additional topics of discussion included risks and benefits of global information-sharing; the vulnerability of global supply chains; the potential need for restructuring and rethinking preparedness metrics under such regimes as the WHO International Health Regulations’ Joint External Evaluations or the Global Health Security Agenda Global Health Security Index; misinformation and disinformation; vulnerabilities in healthcare, laboratory, and biosurveillance infrastructure; insurgent groups using social media and other online platforms to target and recruit susceptible youth; susceptibility to radicalization due to pandemic induced stresses; and apocalyptic thinking, which could stimulate Violent Non-State Actor interest in bioterrorism.

1. Radicci, L, Mandić-Rajčević, S, Masci, F. The COVID-19 Outbreak in Italy: Crisis Management and Medical Experiences. Webinar in: IBPF-EC JRC One Health Security Webinar Series. 07 April 2020. Virtual. [↑](#endnote-ref-1)
2. Bangure, D, Bilounga Ndongo, C, Bruinen de Bruin, Y, Lequarré, A-S. The COVID-19 Response in Africa. Webinar in: IBPF-EC JRC One Health Security Webinar Series. 17 June 2020. Virtual. [↑](#endnote-ref-2)
3. WHO Coronavirus (COVID-19) Dashboard, WHO website. [https://covid19.who.int](https://covid19.who.intu). Accessed 16 June 2021. [↑](#endnote-ref-3)
4. de Bruin YB, Lequarre AS, McCourt J, Clevestig P, Pigazzani F, Jeddi MZ, Colosio C, Goulart M. Initial impacts of global risk mitigation measures taken during the combatting of the COVID-19 pandemic. Safety science. 2020; doi:[10.1016/j.ssci.2020.104773](https://doi.org/10.1016/j.ssci.2020.104773). [↑](#endnote-ref-4)
5. Murray, C. Global COVID-19 Modeling. Webinar in: IBPF-EC JRC One Health Security Webinar Series. 22 July 2020. Virtual. [↑](#endnote-ref-5)
6. Osterhaus A, Vanlangendonck C, Barbeschi M, et al. Make science evolve into a One Health approach to improve health and security: a white paper. 2020. doi:[10.1186/s42522-019-0009-7](https://doi.org/10.1186/s42522-019-0009-7). [↑](#endnote-ref-6)
7. Pillai, S, Jackson, P, Ackerman, R. COVID-19 Epidemiology and Evolution. Webinar in: IBPF-EC JRC One Health Security Webinar Series. 26 August 2020. Virtual. [↑](#endnote-ref-7)
8. SARS-CoV-2 Variant – United Kingdom of Great Britain and Northern Ireland, WHO website. <https://www.who.int/emergencies/emergency-events/item/2020-DON304>. Accessed 16 June 2021. [↑](#endnote-ref-8)
9. # Coronavirus Disease 2019 (COVID-19) Science Brief: Emerging SARS-CoV-2 Variants, CDC website. <https://www.cdc.gov/coronavirus/2019-ncov/science/science-briefs/scientific-brief-emerging-variants.html>. Accessed 16 2021.

   [↑](#endnote-ref-9)
10. SARS-CoV-2 Variant Classifications and Definitions, IBPF website. <https://www.ibpforum.org/reportsandpublications/sarscov2-variant-classifications-and-definitions>. Accessed 18 June 2021. [↑](#endnote-ref-10)
11. Gawlik, B, Good, S, Luwaga, L. COVID-19 Environmental Surveillance and Risk Communications. Webinar in: IBPF-EC JRC One Health Security Webinar Series. 23 September 2020. Virtual. [↑](#endnote-ref-11)
12. de Wijk, R, Welle, N, Ackerman, G. What Comes Next?: Security Implications Post-COVID World. Webinar in: IBPF-EC JRC One Health Security Webinar Series. 27 January 20210. Virtual. [↑](#endnote-ref-12)
